# Hard and Soft Tissue Dimensional Changes Following Tooth Extraction: A Clinical and Radiological Pilot Study

**DOI:** 10.7759/cureus.111736

**Published:** 2026-06-29

**Authors:** Julie Rohban, Abdallah Menhall, Pierre Lahoud, Nabil Ghosn, Christian Makary

**Affiliations:** 1 Department of Oral Surgery, Faculty of Dental Medicine, Saint Joseph University of Beirut, Beirut, LBN

**Keywords:** bone resorption, digital dentistry, dimensional alterations, extraction sites, post-extraction ridge alteration, soft tissue retraction

## Abstract

Objectives: This clinical and radiological pilot study aimed to evaluate soft and hard tissue dimensional changes at extraction sites using digital technology.

Methods: Ten teeth were extracted from seven patients referred for simple tooth extractions. Cone-beam computed tomography (CBCT) scans and digital impressions were obtained before tooth extraction and four months after extraction. Virtual models were initially aligned using Medit® Design software (Medit, Seoul, Republic of Korea) and subsequently superimposed onto CBCT images using BlueSkyPlan® software (Blue Sky Bio, LLC, Grayslake, IL, USA). Horizontal and vertical measurements of soft and hard tissue dimensional changes were performed using BlueSkyPlan®. Vertical measurements were obtained at the buccal, central socket, and palatal/lingual aspects. Horizontal measurements were taken at four levels, 2 mm apart, starting at the initial ridge level (L0).

Results: Horizontal gingival retraction was 27.40% at L0 and 11.58% at L2. Horizontal hard tissue resorption was 24.39% at L0 and 30.09% at L2. A significant difference in mean soft tissue thickness (at L0 and L2) and hard tissue thickness (at all levels) was observed before and after extraction (p < 0.05). The mean vertical soft tissue retraction was 0.94 ± 0.48 mm buccally and 1.21 ± 0.93 mm lingually. Mean vertical hard tissue resorption was 1.92 ± 1.23 mm buccally and 1.71 ± 0.88 mm lingually. However, no significant differences were observed between buccal and palatal/lingual vertical soft and hard tissue dimensional changes.

Conclusion: This study showed that extensive bone resorption and gingival retraction generally occur within four months after tooth extraction. These post-extraction dimensional changes should be considered when establishing surgical and prosthetic treatment plans.

## Introduction

Tooth preservation is achieved through preventive, restorative, endodontic, and periodontal treatments aimed at controlling disease and maintaining tooth-supporting structure [1]. However, it is sometimes impossible to preserve them, and therefore, their extraction is considered [1,2]. Following tooth removal, biological events leading to socket healing occur [3]. These biological events are divided into four successive phases: hemostasis/coagulation, inflammation, proliferation, and tissue modeling/remodeling [4].

Horizontal and vertical dimensional changes occur in both hard and soft tissues [3,5]. Observed bone resorption is more pronounced horizontally than vertically [3,6]. Additionally, vertical bone resorption is more pronounced on the buccal side than interproximally and is also more intense buccally compared to the palatal or lingual side [3,7].

Dimensional changes are also observed in soft tissue, and during the weeks following extraction, soft tissue increases in volume and covers the socket, owing to quick cell proliferation [8,9]. These post-extraction dimensional changes are influenced by patient-related factors (smoking habits, diabetes), extraction site (molar or other), gingival biotype, and type of surgery (flap elevation, simple extractions) [3]. Practitioners must consider all these post-extraction dimensional alterations to be able to establish a proper treatment plan, especially when planning implant placement or prosthetic rehabilitation [3].

While two-dimensional radiographs, such as periapical and panoramic X-rays, were used to follow-up socket resorption, cone-beam computed tomography (CBCT) is now considered the best technique to precisely measure bone resorption at extraction sites [10]. It is a powerful device that provides three-dimensional images and allows for various views: panoramic, sagittal/vertical, coronal, and axial [11,12].

Clinical measurements of ridge dimensional changes were mainly recorded with a periodontal probe on casts following conventional impression [6]. Vertical and horizontal changes could therefore be evaluated on plaster models following irreversible hydrocolloid or silicone impression performed prior to tooth extraction and then at different timepoints [6]. However, this method has many disadvantages, such as impression material distortion and hygroscopic plaster expansion during pouring, resulting in imprecise plaster models [13].

To address all problems encountered with conventional methods, intra-oral scanners were recently introduced [14]. Intra-oral scanners produce digital impressions by collecting data from patient's mouth and converting it into a virtual model [15,16]. Intra-oral scanners were recently used to measure post-extraction resorption and are considered a valid method [17,18].

Digital impressions can be combined with CBCT scans to evaluate post-extraction changes. Digital impressions are exported as Standard Tessellation Language or Stereolithography (STL) files, while CBCT scans are saved in the Digital Imaging and Communication in Medicine (DICOM) format [18]. These files are imported into implant planning software, where they are superimposed following different reference points for maximum precision, hence allowing precise and detailed hard and soft tissue structure visualization [19]. This technology facilitates ridge dimensional change analysis and optimizes implant positioning, enhancing precision and predictability in treatment outcomes.

This study aimed to assess four-month post-extraction dimensional changes in soft and hard tissues using digital technology.

## Materials and methods

This study was conducted at the Faculty of Dental Medicine of Saint Joseph University of Beirut, between September 2022 and March 2023. The research protocol was approved by the Institutional Ethics Committee (Tfemd/2023/49). All participants were informed about the study, and written informed consent was obtained prior to enrollment.

Study population 

Seven patients (mean age: 48 years), comprising four females and three males, were recruited from the Department of Oral Surgery at Saint Joseph University of Beirut. Patients were referred for an extraction of single- or double-rooted teeth in the maxilla or mandible, followed by implant-supported rehabilitation. Inclusion criteria consisted of adult patients (≥18 years) requiring extraction of posterior maxillary or mandibular teeth, with planned implant placement and intact socket walls suitable for clinical and radiographic assessment after extraction. Patients with systemic diseases or conditions affecting bone healing, active periodontal disease, smoking habits, non-intact socket walls, or when grafting or socket preservation procedures were indicated or performed were excluded. A total of 10 posterior teeth were extracted, with an equal distribution between the mandible (n = 5) and maxilla (n = 5). The indications for extraction included major crown fractures, prosthetic considerations, and deep carious lesions. All extraction sites were allowed to heal spontaneously without bone grafting, socket preservation, or ridge preservation procedures.

This study was designed as a pilot investigation, with the primary objective of assessing the feasibility and applicability of the digital superimposition workflow for evaluating dimensional changes following tooth extraction. Owing to the exploratory nature of the study and the limited number of eligible participants available during the study period, a formal sample size calculation was not performed. The data generated are intended to provide preliminary information and support the design of future studies with larger cohorts, rather than to test definitive hypotheses.

Clinical procedure

A representative patient included in this study was a 40-year-old male, a non-smoker, with no systemic diseases. He was referred for an extraction of the first maxillary premolar, with planned subsequent implant placement. Oral hygiene was good, and no periodontal pathology was present. Prior to extraction, a digital impression (3 Shape Trios Intra-Oral Scanner) was obtained, and a CBCT scan was performed (NewTom VGI, QR srl, Verona, Italy). Under local anesthesia, the tooth was atraumatically extracted using forceps in order to preserve the socket walls. No bone grafting or socket preservation procedures were performed. Four months after extraction, the patient was recalled, and clinical and radiographic evaluations were repeated according to the study protocol.

Linear Measurements of Dimensional Changes in Soft and Hard Tissue

**Figure 1:**
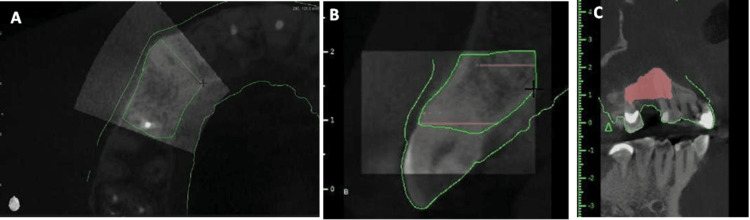
Extraction site segmentation at T1: (A) axial view, (B) sagittal view, (C) segmentation completed

**Figure 2:**
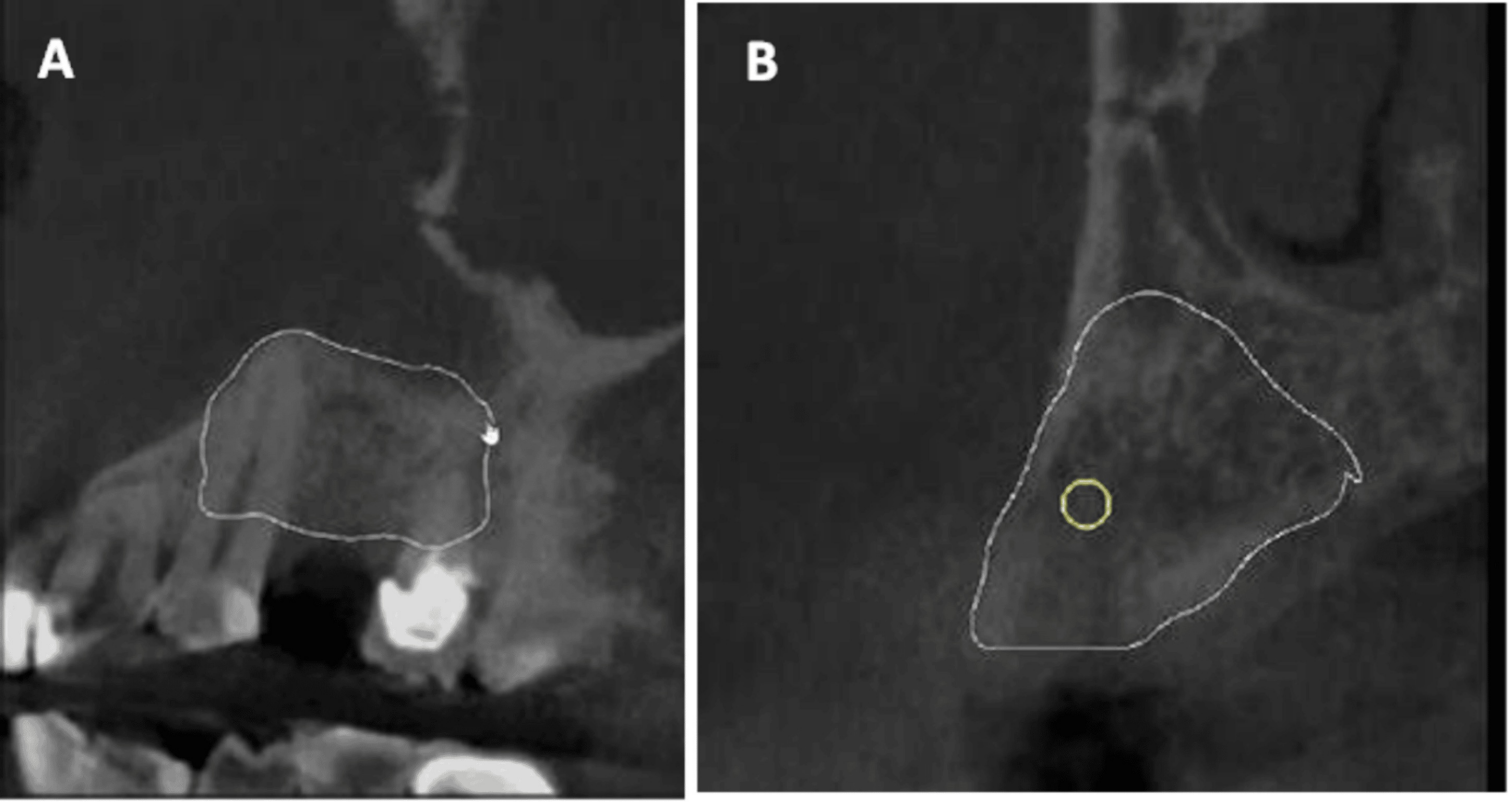
Final extraction socket at T1: (A) panoramic view, (B) sagittal view

Horizontal and vertical gingival retraction and bone resorption were measured on BlueSkyPlan® software (Blue Sky Bio, LLC, Grayslake, IL, USA). The following methodology was used: first, the post-extraction (T1) CBCT scan was imported to BlueSkyPlan® as a DICOM file. The extraction site was segmented into multiple parts to achieve the final bone contour (Figures 1, 2). 

The same procedure was performed on pre-extraction (T0) CBCT scans. The initial pre-extraction socket bone contour was obtained after segmentation.

**Figure 3:**
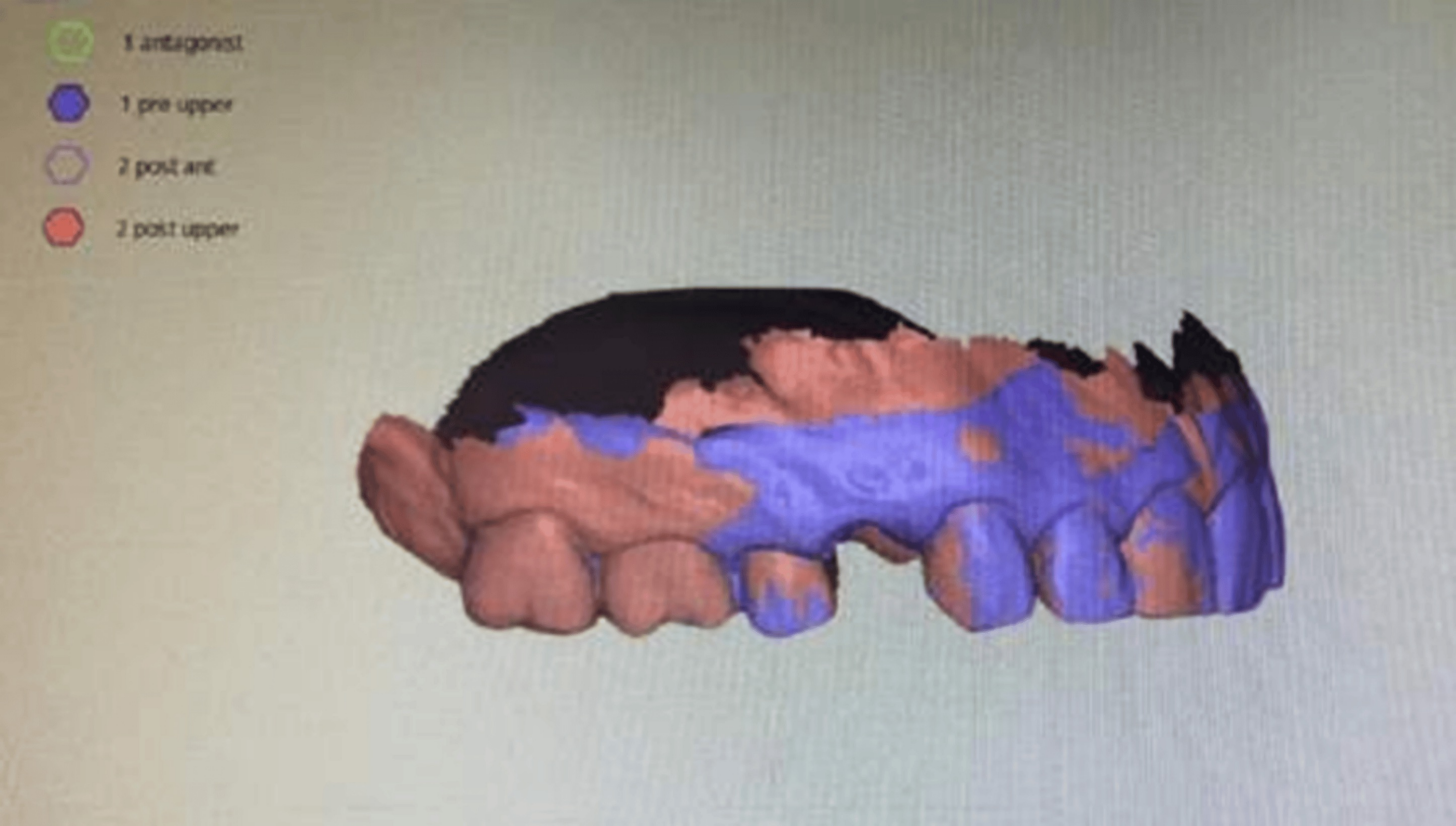
Virtual model alignment and superimposition at T0 (purple) and T1 (pink) on the Medit® (Medit, Seoul, Republic of Korea) design software

Then, pre-extraction and four-month post-extraction virtual models were imported to the Medit® design platform (Medit Link v 2.4.4; Medit, Seoul, Republic of Korea) as STL files, where they were spatially aligned and automatically superimposed following the buccal surfaces of adjacent and posterior teeth located outside the surgical site as reference landmarks (Figure 3). These structures were selected because they represent stable hard-tissue surfaces that are not expected to undergo dimensional changes following tooth extraction and healing, thereby providing reliable reference areas for registration and superimposition.

**Figure 4:**
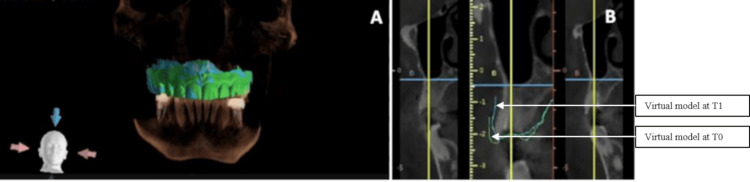
Virtual model superimposition with pre-extraction CBCT: (A) 3D view showing virtual model at T0 (green) and virtual model at T1 (blue), (B) sagittal view

STL files were then transferred to BlueSkyPlan®, where they were automatically superimposed with DICOM files, following buccal reference points, using the software’s surface-based registration algorithm and resulting in four superimposed contours: pre- and post-extraction gingival and bone contours (Figure 4). Following registration, the accuracy of superimposition was visually verified to ensure optimal matching of the selected reference surfaces. Care was taken to avoid movable soft-tissue regions and areas potentially affected by post-extraction remodeling, thereby minimizing registration errors.

**Figure 5:**
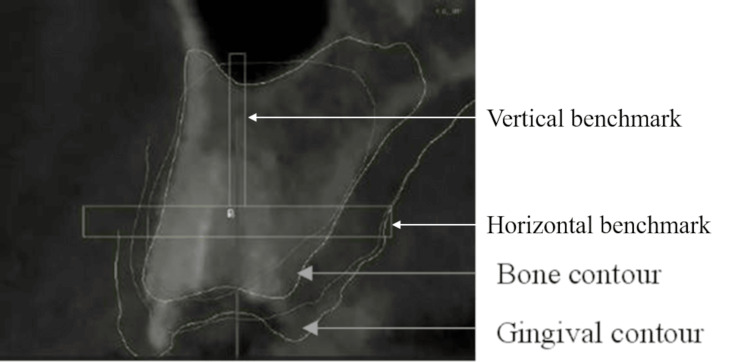
CBCT sagittal section at T0 showing four contours: bone and gingival contours at T0 (green) and T1 (orange), vertical virtual benchmark (pink), and horizontal virtual benchmark (yellow)

Virtual vertical and horizontal benchmarks were then inserted (Figure 5). The vertical reference line represented the planned long axis of the future implant, while the horizontal reference line was constructed perpendicular to this axis.

**Figure 6:**
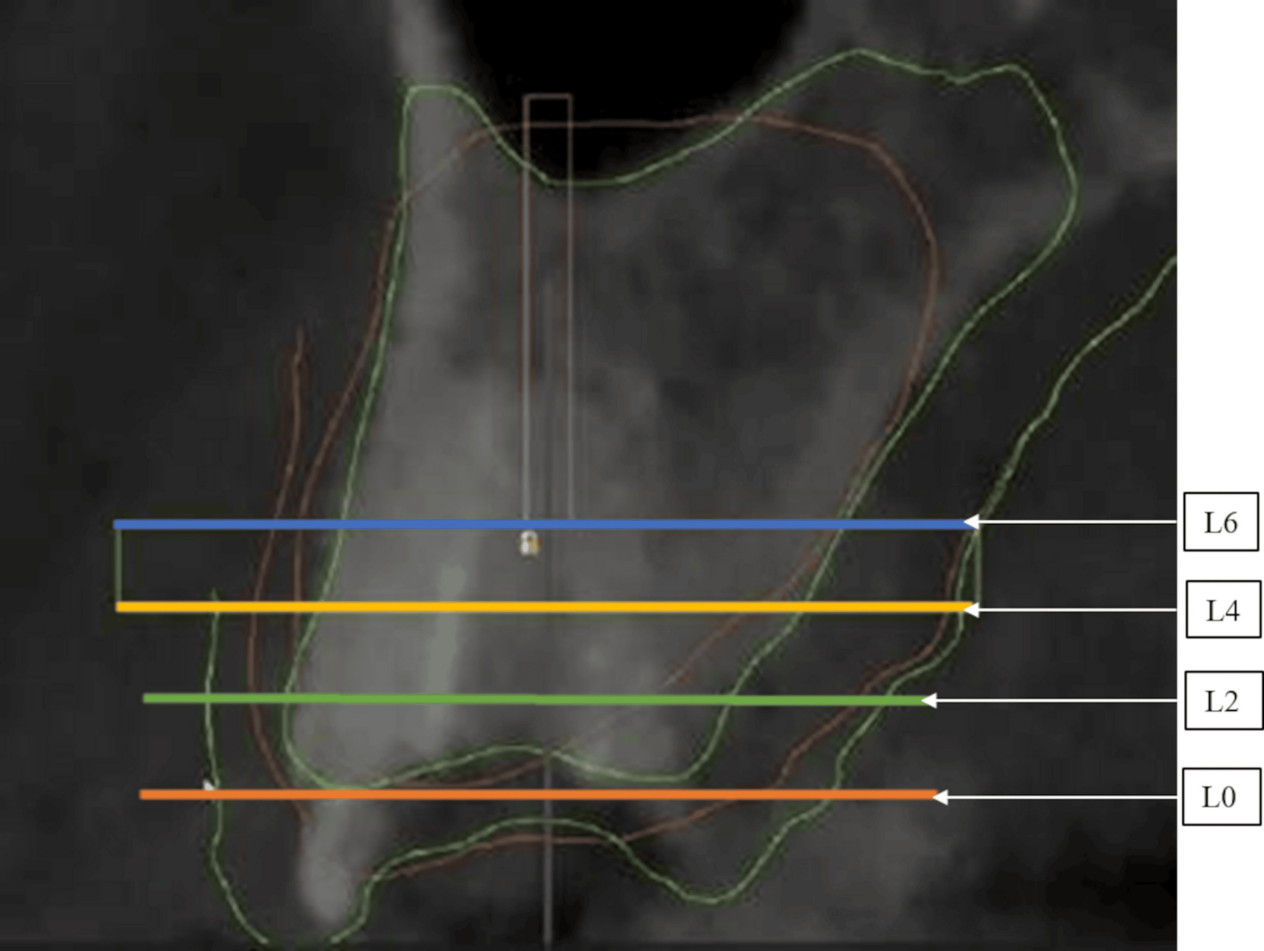
Different levels used to perform the measurements: L0 (orange), L2 (green), L4 (yellow), and L6 (blue)

**Figure 7:**
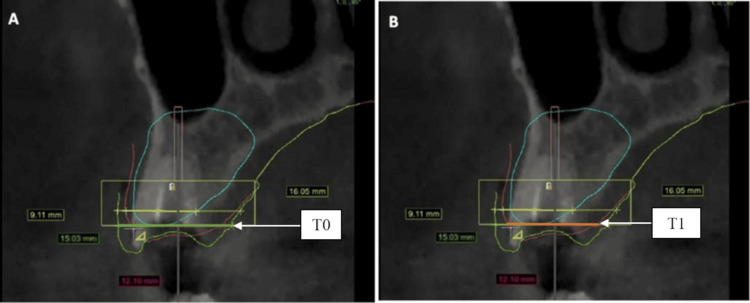
Horizontal measurements performed on soft tissue at L0: (A) horizontal measurement (15.03 mm) of soft tissue contour at T0 (green), (B) horizontal measurement (12.10 mm) of soft tissue contour at T1 (orange)

Horizontal soft and hard tissue measurements were taken at four levels, with 2-mm intervals, with the first level (L0) defined as the initial ridge level (Figures 6, 7).

**Figure 8:**
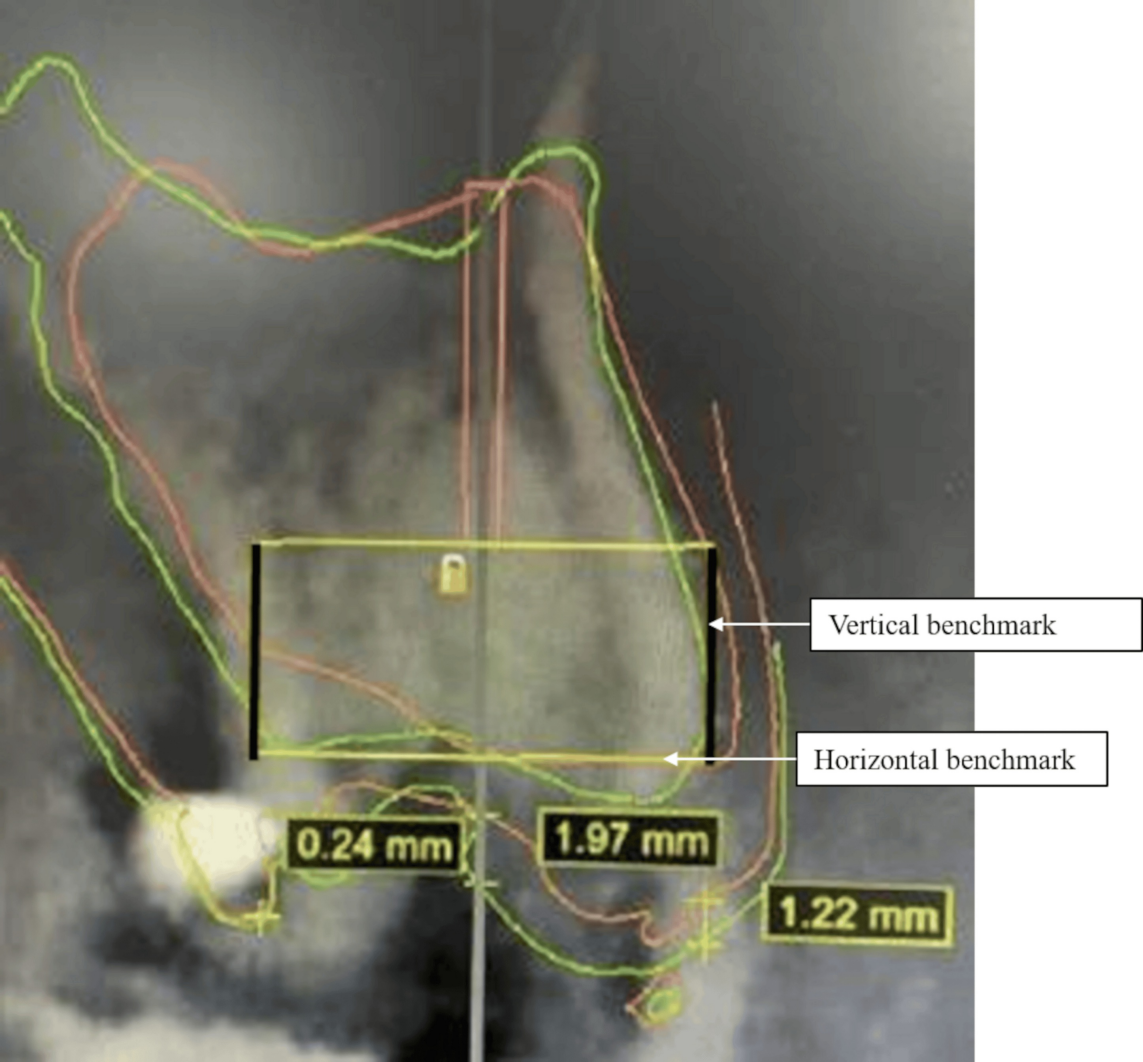
Vertical measurements performed on soft tissue: soft tissue contour at T0 (green) and T1 (orange), horizontal benchmark positioned at L0 (yellow), vertical benchmark axis (black)

Vertical soft and hard tissue measurements were performed buccally, at the center of the socket, and palatally/lingually, using the same benchmarks. The horizontal benchmark was positioned at the initial alveolar crest level (L0). Bone resorption and gingival retraction were measured vertically along the vertical horizontal benchmark axis at three positions (Figure 8).

## Results

Statistical analysis

A descriptive analysis was conducted. Variables were all quantitative and described by their means and standard deviations (SD). Normality was checked using the Kolmogorov-Smirnov test. The paired-sample T test was used to compare different horizontal and vertical means. Statistical significance was set at p-value ≤ 0.05. Analysis was performed using IBM SPSS Statistics for Windows, Version 25.0 (Released 2017; IBM Corp., Armonk, NY, USA).

Values obtained in mm before and after extraction were compared for soft (Table 1) and hard tissue (Table 2).

**Table 1:** Soft tissue horizontal measurements (in mm) before and after extraction (n = 10)

Level	Before extraction		After extraction		T-test value	p-value
	Mean	SD	Mean	SD		
0	10.84	3.03	8.19	3.28	8.917	<0.001
2	11.63	3.02	10.29	2.87	4.340	0.002
4	12.59	3.51	11.69	2.72	2.064	0.073
6	13.64	3.67	13.00	3.04	1.466	0.186

**Table 2:** Hard tissue horizontal measurements (in mm) before and after extraction (n = 10)

Level	Before extraction		After extraction		T-test value	p-value
	Mean	SD	Mean	SD		
0	7.98	1.60	6.16	2.07	7.474	<0.001
2	8.62	1.65	6.07	1.60	8.575	<0.001
4	9.15	1.63	7.65	1.49	6.595	<0.001
6	9.31	1.77	8.09	2.03	3.079	0.013

There was a significant difference in average soft tissue thickness at L0 (t = 8.917, p < 0.001) and L2 (t = 4.340, p = 0.002). Right before extraction, at L0, average soft tissue thickness was 10.84 ± 3.03 mm. At four months after extraction, at L0, average soft tissue thickness was 8.19 ± 3.28 mm. In addition, there was a significant difference between average hard tissue thickness before and after extraction at all levels: L0 (t = 7.474, p < 0.001), L2 (t = 8.575, p < 0.001), L4 (t = 6.595, p < 0.001), and L6 (t = 3.079, p = 0.013). Before extraction, the average bone thickness at L0 was 7.98 ± 1.60 mm and 6.16 ± 2.07 mm at four months.

Moreover, horizontal gingival retraction was 27.40% at L0 and 11.58% at L2. Horizontal hard tissue resorption was 24.39% at L0 and 30.09% at L2 (Table 3). There was a significant difference between average soft tissue thickness before and after extraction at L0 and L2, as well as average hard tissue thickness at all levels (p < 0.05).

**Table 3:** Horizontal bone resorption percentage and horizontal gingival retraction percentage (n = 10)

Level	Soft tissue		Hard tissue	
	Mean	SD	Mean	SD
0	27.40	16.35	24.39	11.72
2	11.58	9.88	30.09	10.51
4	5.67	10.59	16.34	6.92
6	3.34	10.70	13.14	11.26

As for vertical dimensional changes (Table 4), mean vertical buccal soft tissue retraction was 0.94 ± 0.48 mm, and palatal/lingual retraction was 1.21 ± 0.93 mm. For hard tissue, mean vertical resorption was 1.92 ± 1.23 mm buccally and 1.71 ± 0.88 mm palatally/lingually. However, there was no significant difference between buccal and palatal soft and hard tissue dimensional changes.

**Table 4:** Mean vertical bone resorption and gingival retraction in mm (n = 10) ^a^p-value of test comparing buccal and palatal soft tissue vertical retraction. ^b^p-value of test comparing buccal and palatal hard tissue vertical resorption.

Level	Soft tissue		Hard tissue	
	Mean	SD	Mean	SD
Buccal	0.94	0.48	1.92	1.23
Mid	1.03	0.46	1.36	1.24
Palatal	1.21	0.93	1.71	0.88
T-test buccal-palatal	-1.021		0.595	
P-value buccal-palatal	0.337^a^		0.566^b^	

## Discussion

This study aimed to assess four-month post-extraction dimensional changes in soft and hard tissues using digital technology.

Tooth extraction is a common procedure in oral surgery that initiates biological remodeling of the alveolar ridge, resulting in variable degrees of horizontal and vertical hard and soft tissue alterations [20]. These changes may compromise subsequent prosthetic and implant rehabilitation, particularly in esthetic zones. Post-extraction remodeling is influenced by both surgical and biological factors [21]. Surgical trauma, including flap elevation, has been associated with increased bone loss compared with atraumatic and flapless techniques [21,22]. In the present study, all extractions were performed atraumatically and flapless to minimize procedure-related bone alterations. In addition, patient-related factors such as gingival biotype and buccal bone thickness significantly influence the magnitude of ridge resorption [3]. Thin buccal bone (≤1 mm) and thin periodontal phenotype have been associated with greater vertical and horizontal bone loss compared with thicker biotypes [8,23,24]. However, these variables were not measured in the present study, which may limit direct comparison with existing literature.

Accurate assessment of these dimensional changes is essential for implant planning and treatment decision-making [3]. With the advancement of digital dentistry, highly precise and reproducible tools are now available for such evaluations [11]. In this study, intraoral scanning and CBCT imaging were combined using implant planning software, allowing superimposition of STL and DICOM datasets for three-dimensional analysis of hard and soft tissue changes [24]. This digital workflow provides higher accuracy, standardization, and reproducibility compared with conventional methods such as plaster models or manual measurements [15,25,26]. Although precautions were taken during model registration, a certain degree of superimposition error is inherent to digital alignment procedures. Consequently, minor inaccuracies in registration may have influenced the measured dimensional changes and should be considered when interpreting the results. CBCT imaging, considered a reference modality in implant dentistry, further enables detailed three-dimensional assessment of hard tissue changes with relatively low radiation exposure [11,12,23].

Tooth extraction socket management strategies have been widely investigated to reduce post-extraction dimensional changes. Techniques such as immediate implant placement, socket grafting, and membrane-assisted ridge preservation have been proposed to maintain ridge dimensions [27]. Ridge preservation procedures have demonstrated a significant reduction in post-extraction bone loss compared with extraction alone and may better maintain ridge height and width [27,28]. However, residual resorption still occurs, with studies reporting approximately 1.7 mm horizontal bone loss even in preserved sites, indicating that complete preservation of the original ridge contour is not achievable [29]. Moreover, continued buccal bone remodeling has been reported even after immediate implant placement with simultaneous grafting [18]. In the present study, no ridge preservation procedures were performed, allowing evaluation of physiological socket healing and natural post-extraction remodeling.

The timing of post-extraction assessment is clinically relevant, as the majority of early dimensional changes occur within the first 3-4 months of healing [29]. Therefore, a four-month follow-up period was selected in this study to evaluate early remodeling at a clinically relevant stage for implant planning. This timeframe is consistent with previously reported healing intervals of 3-6 months in the literature and allows sufficient bone maturation for reliable radiographic evaluation [29].

The results of the present study demonstrated a significant decrease in bone volume and tissue contour at extraction sites. Four months postoperatively, at L0, a horizontal gingival contour retraction of 2.65 ± 0.25 mm was recorded. A similar study measured soft tissue alterations four months post-extraction in the anterior region, using STL data obtained from scanned cast models. At four months, soft tissue volume decreased by 2.60 ± 0.32 mm at L2 [30]. This demonstrates that soft tissue follows alveolar bone dimensional changes during the healing process. In the present study, average vertical gingival retraction was 0.94 ± 0.48 mm buccally and 1.21±0.93 mm palatally/lingually. These results agree with those of another study that found vertical soft tissue loss < 1 mm after a period of 12 months [6]. In our study, there was no significant difference between buccal and palatal soft tissue vertical dimensional changes. This can be due to our limited sample size, as this could have reduced the statistical power to detect a significant difference. Moreover, gingival biotype, a factor not evaluated in our study, may have influenced these results. If soft tissue thickness were similar on the buccal and palatal sides, this might have contributed to uniform healing [31]. Furthermore, soft tissue healing can vary significantly from one individual to another, depending on factors like blood supply or immune system function. The impact of soft tissue healing on dimensional changes in post-extraction sites has received little attention in the literature. Therefore, further clinical studies with larger samples should be conducted to better assess gingival retraction at extraction sites, taking into consideration the patient's initial gingival biotype and buccal bone thickness.

Since soft tissue contours largely depend on the underlying bone structure, hard tissue resorption was also assessed. At L0, 24.39% horizontal bone resorption was observed, and 30.09% at L2. Horizontal bone loss was measured in terms of percentage because it is clinically more relevant. These results agree with other studies showing horizontal bone loss ranging from 32% at three months, and 29%-63% at 6-7 months post-extraction [3,30]. They are in accordance with the results of another study that superimposed DICOM data of two CBCT sections to perform measurements and showed a horizontal bone resorption of 30.62% at L4, at four months post-extraction [19]. Horizontal bone resorption observed in our study, as well as in the literature, is an expected result of tooth extraction and is an inherent condition of the socket healing process [4,24]. Studies have shown that <65% of dimensional alterations happen within the first three months post-extraction [6]. This is due to high osteoclastic activity during the first eight weeks following extraction [4]. In our study, average vertical buccal and lingual bone resorption were 1.92 ± 1.23 mm and 1.71 ± 0.88 mm, respectively. A similar study measured vertical bone resorption at four months. They reported 2.1 ± 0.6 mm resorption buccally and 2 ± 0.73 mm lingually at four months post-extraction [27]. In another study also using digital dentistry methods and superimposed CBCTs on implant planning software, mean vertical mid-facial and mid-palatal bone reduction were 2.17 ± 2.70 mm and 1.48 ± 1.56 mm, respectively, at 14 weeks post-extraction [32].

While previous studies have shown that alveolar ridge vertical resorption is more pronounced buccally than palatally/lingually, our study was unable to demonstrate this [6,32]. Early bundle bone resorption may partially explain the marked buccal plate resorption [3]. In fact, teeth are attached to the jawbone through the bundle bone, into which periodontal ligament fibers are embedded. The buccal bone is largely composed of bundle bone. Since bundle bone is a tooth-dependent entity, it will rapidly resorb after extraction, leading to the loss of a significant vestibular plate portion [3]. Preoperative buccal and palatal bone thickness measurements were not considered in this study and could be a reason for this non-significant difference. Other clinical studies with larger samples should be conducted to explore the potential influence of initial buccal and palatal bone thickness on vertical resorption patterns.

The present study included a limited sample size and should therefore be considered exploratory. While the findings provide preliminary insights into post-extraction dimensional changes, larger prospective studies are required to confirm these observations and improve the generalizability of the results. Moreover, multiple statistical comparisons were performed across several measurement levels without adjustment for multiple testing. Consequently, the possibility of a type I error cannot be excluded. Furthermore, because multiple extraction sites were obtained from some patients, the independence of observations may have been partially compromised by clustering effects. Future studies with larger sample sizes should consider statistical approaches that account for repeated measurements and within-patient correlations. A further limitation is the absence of intra-observer and inter-observer reliability assessments. Because digital superimposition constituted the primary measurement methodology, evaluation of measurement reproducibility would have strengthened the validity of the findings. Future investigations should include repeated measurements and calculation of reliability indices such as intraclass correlation coefficients.

## Conclusions

In conclusion, this clinical and radiological pilot study demonstrated significant bone resorption and gingival recession four months following tooth extraction. The use of digital technologies enabled precise and reproducible measurements, thereby enhancing the reliability of the results. Intraoral scanners and implant planning software allow for more accurate, efficient, and standardized clinical assessments.

Digital dentistry may further expand the scope of such analyses. In addition to linear measurements, future studies could incorporate volumetric assessments to provide a more comprehensive evaluation of post-extraction dimensional changes.
